# Genomic and Transcriptomic Alterations Associated with STAT3 Activation in Head and Neck Cancer

**DOI:** 10.1371/journal.pone.0166185

**Published:** 2016-11-17

**Authors:** Noah D. Peyser, Kelsey Pendleton, William E. Gooding, Vivian W. Y. Lui, Daniel E. Johnson, Jennifer R. Grandis

**Affiliations:** 1 Department of Otolaryngology–Head and Neck Surgery, University of California San Francisco, San Francisco, CA, United States of America, 94143; 2 Department of Otolaryngology, University of Pittsburgh and the University of Pittsburgh Cancer Institute, Pittsburgh, PA, United States of America, 15213; 3 Biostatistics Facility, University of Pittsburgh Cancer Institute, Pittsburgh, PA, United States of America, 15213; 4 Department of Pharmacology and Pharmacy, School of Biomedical Sciences, Li-Ka Shing Faculty of Medicine, the University of Hong Kong, Hong Kong SAR, China; 5 Department of Medicine, University of Pittsburgh and the University of Pittsburgh Cancer Institute, Pittsburgh, PA, United States of America; Virginia Commonwealth University, UNITED STATES

## Abstract

**Background:**

Hyperactivation of STAT3 via constitutive phosphorylation of tyrosine 705 (Y705) is common in most human cancers, including head and neck squamous carcinoma (HNSCC). STAT3 is rarely mutated in cancer and the (epi)genetic alterations that lead to STAT3 activation are incompletely understood. Here we used an unbiased approach to identify genomic and epigenomic changes associated with pSTAT3(Y705) expression using data generated by The Cancer Genome Atlas (TCGA).

**Methods and Findings:**

Mutation, mRNA expression, promoter methylation, and copy number alteration data were extracted from TCGA and examined in the context of pSTAT3(Y705) protein expression. mRNA expression levels of 1279 genes were found to be associated with pSTAT3(705) expression. Association of pSTAT3(Y705) expression with caspase-8 mRNA expression was validated by immunoblot analysis in HNSCC cells. Mutation, promoter hypermethylation, and copy number alteration of any gene were not significantly associated with increased pSTAT3(Y705) protein expression.

**Conclusions:**

These cumulative results suggest that unbiased approaches may be useful in identifying the molecular underpinnings of oncogenic signaling, including STAT3 activation, in HNSCC. Larger datasets will likely be necessary to elucidate signaling consequences of infrequent alterations.

## Introduction

Head and neck squamous cell carcinoma (HNSCC) is a common and frequently lethal cancer. Recent studies have elucidated the genetic landscape of HNSCC and demonstrated that mutational activation of oncogenic drivers is uncommon in HNSCC. [1–3] The number of mutations in an individual tumor ranged from 3 to 1433 with a median of 103 in a recent report from The Cancer Genome Atlas (TCGA). [3] This genomic heterogeneity underscores the challenges in developing targeted molecular therapies for HNSCC treatment. To date, the epidermal growth factor receptor-directed monoclonal antibody cetuximab is the only molecularly targeted agent that is FDA-approved for the treatment of HNSCC, though clinical responses to cetuximab remain modest and predictive biomarkers are undefined. [4] Examination of oncogenic signaling pathways rather than any single genetic variant may be of use to elucidate the molecular underpinnings of HNSCC. [5,6] Among the most common signaling aberrations in HNSCC is constitutive activation of signal transducer and activator of transcription-3 (STAT3).

STAT proteins comprise a family of transcription factors that transmit cytokine and growth factor stimuli from cell surface receptors to the nucleus, leading to induction of a wide array of genes involved in a multitude of normal and oncogenic cellular functions. Seven members of the STAT protein family have been identified: STAT1-4, STAT5a, STAT5b, and STAT6, each of which contains a DNA binding domain, a Src-homology 2 (SH2) domain, and a key tyrosine residue that is essential for activation. [7] Phosphorylation of STAT3 on tyrosine 705 (Y705) leads to robust pathway activation, and pSTAT3(Y705) expression represents a surrogate marker for active STAT3 signaling. STATs were first implicated in mammalian cell oncogenesis when Src oncogene-transformed cells were found to express constitutively active STAT3. [8] Furthermore, STAT3 activation has been identified as a requirement for Src-mediated transformation [9], and constitutively active STAT3 was found to mediate transformation of immortalized fibroblasts, leading to the recognition of STAT3 as a bona fide oncoprotein. [10] Aberrant activation of STAT3 has been detected in a variety of cancers, including breast, ovarian, prostate, multiple myeloma, leukemias, lymphomas, and HNSCC, among others. [11]

Although many upstream kinases that activate STAT3 via Y705 phosphorylation have been defined, the genetic alterations associated with constitutive STAT3 phosphorylation and activation in HNSCC remain incompletely understood. The detailed information amassed by The Cancer Genome Atlas (TCGA) provides an opportunity to interrogate the alterations that are associated with increased expression of phospho-proteins assessed in The Cancer Proteome Atlas (TCPA), including pSTAT3(Y705), in an unbiased manner. In the present study, we analyzed TCGA and TCPA data to identify genetic or epigenetic alterations, including somatic mutation, promoter methylation, mRNA expression, and copy number alteration, which are associated with elevated pSTAT3(Y705) expression in HNSCC in order to identify events that contribute to STAT3 activation in this malignancy.

## Methods

### Computational Analyses and Statistics

HNSCC tumor data were retrieved from The Cancer Genome or Proteome Atlas. Our cohort contained 206 HNSCC primary tumors with expression analysis of 200 proteins and phospho-proteins, including pSTAT3(Y705), as assessed by reverse phase protein array (RPPA). For each tumor, whole exome sequencing, DNA methylation (Illumina Infinium HM450 assay), mRNA expression (RNASeq V2), and copy number alteration (Affymetrix SNP 6.0 array, interpreted using the GISTIC algorithm) data were retrieved from the TCGA Data Matrix. RPPA data were retrieved from The Cancer Proteome Atlas. [12]

Categorical variables (mutation and hypermethylation) were analyzed using Wilcoxon tests to assess a difference in pSTAT3(Y705) expression between mutated/hypermethylated compared with wild-type/non-hypermethylated. As previously described, hypermethylation was defined as a level of methylation for a genetic locus that is at least three standard deviations greater than the mean methylation of the same locus in organ-matched normal tissue as determined by the TCGA. [13] For continuous variables such as mRNA expression, Spearman’s correlation test was used to establish whether mRNA expression and pSTAT3(Y705) protein expression were correlated. For copy number alteration, an ordinal variable, a two-tailed Jonckheere-Terpstra statistic was calculated for each region. For all studies a false discovery rate (FDR) < 0.1 was considered significant. Expected false discovery rates were based on the q value [14] and were computed with the R package qvalue. [15]

### Cells, Reagents, and Immunoblotting

UMSCC-47 cells (obtained from Dr. Thomas E. Carey, University of Michigan) stably expressing WT caspase-8 or vector control were grown in Dulbecco's Modified Eagle's Medium (Corning, Corning, NY, USA) supplemented with 10% fetal bovine serum (Gemini Bio-Products, West Sacramento, CA, USA), 500 μg/mL G418 (Life Technologies, Carlsbad, CA, USA), and 100 units/mL penicillin/streptomycin (Life Technologies). Western blotting was performed with standard methodology. Primary antibodies were directed against pSTAT3(Y705), total STAT3, and caspase-8 (Cell Signaling Technology, Danvers, MA) and were normalized to β-tubulin (Abcam, Cambridge, MA) loading control. Densitometry was performed using ImageJ software.

## Results

### Patient Characteristics

206 primary HNSCC tumors with available pSTAT3(Y705) RPPA data were included in this study (Table 1). Most tumors presented at an advanced stage (64.7% clinical stage III-IV) and were located in the oral cavity (66.0%) or larynx (28.2%). A history of smoking and/or heavy drinking was common (83.5% and 60.7%, respectively). A small number of tumors in this collection were associated with human papilloma virus (HPV) infection (14/200, 7.0%).

**Table 1:** Patient characteristics of 206 HNSCC tumors with RPPA and WES results.

*Characteristics*	*WES Tumors (n = 206)*	* *
***Age*, *years***		
*Median±SD*	*62*	*±12*.*3*
***Sex*, *N (%)***		
*Male*	*149*	*72*.*3%*
*Female*	*57*	*27*.*7%*
***Risk Factors*, *N (%)***		* *
*Smoking history*	*172*	*83*.*5%*
*Alcohol history*	*125*	*60*.*7%*
HPV infection*	*14*	*7*.*0%*
*Tumor Stage*, N (%)**		* *
*T1*	*12*	*6*.*1%*
*T2*	*58*	*29*.*3%*
*T3*	*50*	*25*.*3%*
*T4a*	*78*	*39*.*4%*
*T4b*	*0*	*0*.*0%*
*Nodal Stage*, N (%)***		* *
*N0*	*71*	*43*.*3%*
*N1*	*21*	*12*.*8%*
*N2a*	*4*	*2*.*4%*
*N2b*	*43*	*26*.*2%*
*N2c*	*25*	*15*.*2%*
***Primary tumor site*, *N (%)***		* *
*Oral Cavity*	*136*	*66*.*0%*
*Oropharynx*	*11*	*5*.*3%*
*Hypopharynx*	*1*	*0*.*5%*
*Larynx*	*58*	*28*.*2%*

*N = 200 tumors assessed for HPV infection.

**N = 198 patients with tumor stage data.

***N = 164 patients with nodal stage data.

#### pSTAT3(Y705) Expression was not Associated with Clinical Characteristics at Presentation

**Fig 1:**
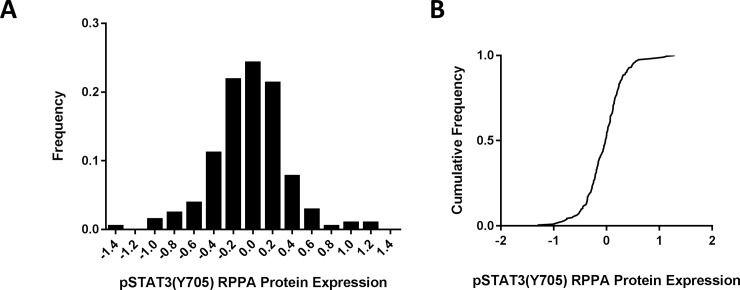
Distribution of pSTAT3(Y705) RPPA scores in HNSCC. The frequency distribution (A) or cumulative frequency (B) of pSTAT3(Y705) normalized RPPA protein expression was plotted for 206 HNSCC tumors. The median for these data was -0.01875 and the range from -1.306 to 1.2934.

RPPA data for pSTAT3(Y705) were retrieved from TCPA and the distribution across 206 tumors was determined (Fig 1). For these tumors, the pSTAT3(Y705) RPPA scores, presented in log_2_ scale, were generally normally distributed with a median of -0.01875 and range from -1.306 to 1.2934. Using univariate analyses, pSTAT3(Y705) protein expression was analyzed with respect to the clinical characteristics summarized in Table 1. No significant difference in pSTAT3(Y705) expression was detected in relation to age, smoking, alcohol consumption, HPV infection, tumor stage, nodal stage, or primary tumor site, suggesting little or no association between clinical characteristics at presentation and STAT3 pathway activation in HNSCC.

#### mRNA Expression of Many Genes was Associated with pSTAT3(Y705) Expression

**Fig 2:**
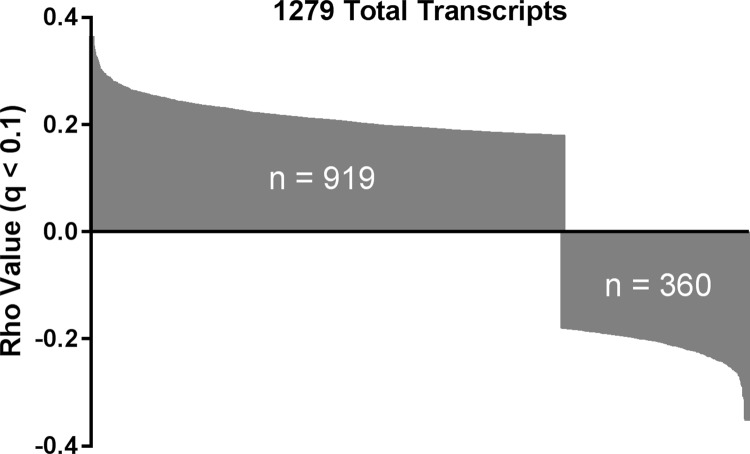
mRNA expression of 1279 genes significantly correlates with pSTAT3(Y705) protein expression. A positive correlation between mRNA expression and pSTAT3(Y705) expression was observed for 919 genes, while a negative correlation was observed for 360 genes.

**Fig 3:**
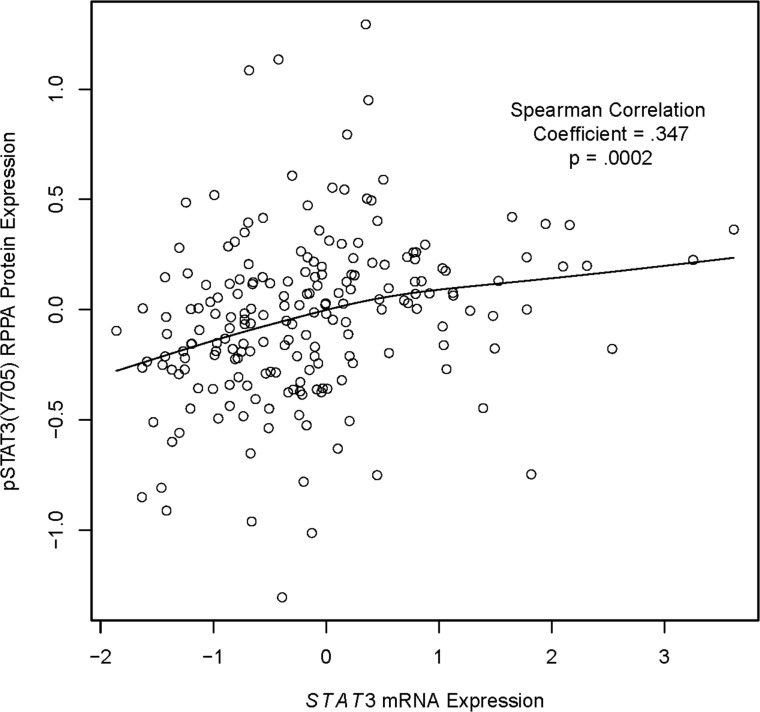
pSTAT3(Y705) expression correlates with *STAT3* mRNA expression. Spearman’s test indicates a significant correlation between *STAT3* mRNA expression and pSTAT3(Y705) protein expression (p = 6.63x10^-7,^, correlation = 0.35).

Protein expression of pSTAT3(Y705) was next analyzed with respect to mRNA expression in HNSCC tumors for 14,789 genes using Spearman’s correlation test. Messenger RNA expression of 1279 of these genes was found to significantly correlate with pSTAT3(Y705) protein expression (q < 0.1) (Fig 2 and S1 Table). The majority of the observed correlations were positive (919/1279, 71.9%), with an increase in pSTAT3(Y705) expression associated with an increase in mRNA expression of the given gene. All correlations were found to be of modest magnitude, with *CX3CR1*, which codes for the CX3C chemokine receptor 1, exhibiting the strongest positive correlation with pSTAT3(Y705) (rho = 0.361; S1 Fig). While *CX3CR1* has been detected in a subset of HNSCC cell lines [16], its oncogenic contribution is largely undetermined. We therefore further filtered statistically significant findings using the COSMIC Cancer Gene Census [17] to prioritize associations of high biological interest. We identified 51 known cancer genes for which mRNA expression significantly correlated with pSTAT3(Y705) expression in HNSCC tumors (S2 Table), of which 88.2% (45/51) were positive correlations. Among these, the greatest absolute correlation coefficient value detected was 0.35 for *STAT3*, indicating that *STAT3* mRNA overexpression may contribute to increased STAT3 phosphorylation in HNSCC (Fig 3). This finding suggests that our unbiased analysis is sufficiently robust to identify gene expression patterns that are likely to affect pSTAT3(Y705) expression in HNSCC.

**Fig 4:**
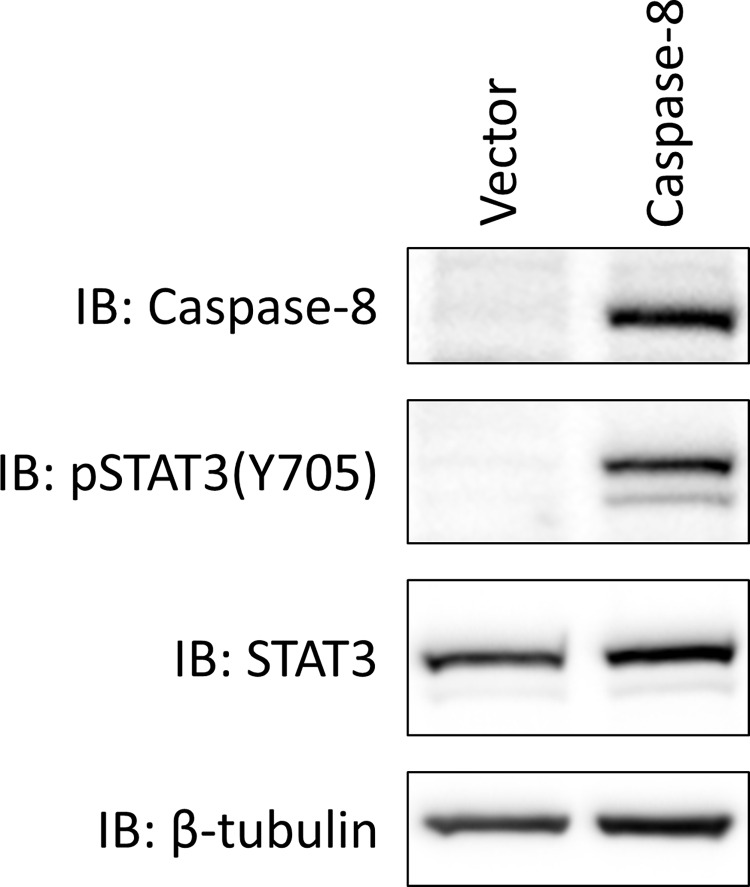
Caspase-8 overexpression is associated with increased pSTAT3(Y705) expression in HNSCC cells. Lysates from UMSCC-47 cells stably expressing caspase-8 or the corresponding vector control were harvested and analyzed for pSTAT3(Y705) expression by immunoblot analysis. The experiment was performed thrice with similar results.

We next sought to evaluate the biologic plausibility of a representative significant association that was mechanistically unexpected. Of particular interest was the observed correlation between caspase-8 mRNA expression and pSTAT3(Y705) expression (rho = 0.283, p = 5.8E-05). Constitutive STAT3 activation inhibits apoptosis. Therefore, it was surprising that expression of a pro-apoptotic gene was associated with pro-oncogenic STAT3 signaling. Furthermore, the *CASP8* gene is altered in 16% of HNSCC tumors (9% of which are mutated) according to The Cancer Genome Atlas. [3] To validate the association between pSTAT3(Y705) and caspase-8 mRNA expression HNSCC cells engineered to overexpress caspase-8 and corresponding vector-transfected controls were analyzed by immunoblot. As shown in Fig 4, stable overexpression of caspase-8 in UMSCC-47 cells was associated with a corresponding increase in pSTAT3(Y705) expression, indicating that our unbiased *in silico* findings may accurately predict unexpected biology. Interestingly, pSTAT3(Y705) expression also strongly correlated with expression of caspase-10 mRNA (S1 Table and S1 Fig). Caspase-8 and -10 are structurally closely related and both act as initiator caspases in the extrinsic apoptosis pathway. The correlation of pSTAT3(Y705) expression with both caspase-8 and -10 hints at a novel functional interaction between STAT3 signaling and mediators of the extrinsic apoptosis pathway.

### Low Frequency Somatic Mutations were Not Associated with pSTAT3(Y705) Expression

We next sought to identify individual genes whose non-synonymous mutation was associated with increased pSTAT3(Y705) expression. Of 14,596 genes analyzed, 7,984 exhibited non-silent mutations in at least 2 HNSCC tumors and were included for further analysis. We then applied Student’s t-test to compare pSTAT3(Y705) expression in wild-type versus mutant tumors for each of these genes and found significant differences for 242 genes (q < 0.1; Table 2). Further analysis indicated that these apparent significant findings are artifacts of low mutation frequency for each gene, indicating insufficiency of the t-test for these data (S2 Fig). We alternatively applied the non-parametric Wilcoxon test and found no genes for which somatic mutation was significantly associated with pSTAT3(Y705) expression (defined as q < 0.1; Table 2).

**Table 2:** Cumulative number of significant results for 7984 genes with 2 or more somatic mutations.

*Test*	*< 0*.*0001*	*< 0*.*001*	*< 0*.*01*	*< 0*.*025*	*< 0*.*05*	*< 0*.*1*	*< 1*
***Student's T***							
*p value*	*137*	*196*	*313*	*451*	*604*	*907*	*7984*
*q value*	*89*	*106*	*155*	*180*	*202*	*242*	*7984*
***Wilcoxon***							
*p value*	*0*	*0*	*34*	*151*	*447*	*939*	*7944*
*q value*	*0*	*0*	*0*	*0*	*0*	*0*	*7802*

### Promoter Methylation and Copy Number Alteration were not Associated with pSTAT3(Y705) Expression

Finally, we examined whether promoter hypermethylation or copy number alteration of any gene or region is significantly associated with increased pSTAT3(Y705) expression. We observed no significant differences in pSTAT3(Y705) protein expression upon hypermethylation of 7,286 genes (S3 Fig), or copy number alteration of 4,309 regions.

## Discussion

STAT3 is frequently hyperactivated by Y705 phosphorylation in HNSCC and other cancers. The determination of somatic alterations that lead to constitutive STAT3 pathway activation may allow for the identification of predictive biomarkers of response to drugs that target STAT3 signaling. In a subset of cancer types, activating mutations of upstream kinases lead to persistent STAT3 pathway activation and predict response to kinase inhibition. Well-characterized examples include the *BCR-ABL* translocation in Philadelphia chromosome-positive leukemias, which confers sensitivity to the ABL tyrosine kinase inhibitor imatinib [18–20], as well as *epidermal growth factor receptor* (*EGFR*)-mutant lung cancers, which are sensitive to EGFR inhibition. [21–25] Additional studies have identified unexpected and low frequency gene mutations that may also lead to pSTAT3(Y705) overexpression, including somatic mutation of *NDUFA13* (encoding GRIM-19; Gene associated with Retinoid Interferon-induced Mortality-19) [26] or *GNAS* (encoding the G_αs_ subunit). [27]

Herein we sought to identify the alterations that are associated with STAT3 pathway activation in HNSCC. Activating kinase mutations are infrequent in HNSCC, indicating that such mutations are unlikely to be of predictive value in this disease, with the notable exception of *PIK3CA* mutation. [6] Instead, recent efforts have investigated the contribution of loss-of-function of negative regulators of STAT3, especially receptor-like protein tyrosine phosphatases. [5,13,28] To date, most efforts have focused on defining phenotypes associated with relatively narrow gene families or pathways of interest in a hypothesis-driven manner, limiting the power to identify associations that would otherwise be unanticipated. For example, previous studies in other cancers have focused on correlations between known pathway components [29], a relatively narrow set of proteins/phospho-proteins [30], and/or make use of only one data type (eg: RNA-Seq). [31] More recent efforts at integrating -omics data for identification of targetable signaling modules have taken divergent approaches, including by multiple hypothesis testing as described here. [32] To our knowledge, a cross-platform and genome-wide search for unexpected alterations that may correlate with increased pSTAT3(Y705) expression in HNSCC has not been reported.

The present study was undertaken to identify putative genomic or epigenomic alterations that lead to STAT3 activation via Y705 phosphorylation in HNSCC. While STAT3 may be alternatively activated by serine 727 (S727) phosphorylation in certain contexts [33,34], the absence of an antibody targeting pSTAT3(S727) in the TCPA array prevents unbiased correlative analysis of this site. Our cohort comprised an atypical North American HNSCC population, in which oral cavity cancers were overrepresented (66%) and oropharyngeal cancers underrepresented (5%). pSTAT3(Y705) expression was not statistically associated with any clinical parameters analyzed including age, sex, tumor site, tumor stage, HPV, smoking, or drinking. We next analyzed mRNA expression data available from TCGA and found that expression of 1279 genes significantly correlated (q < 0.1) with pSTAT3(Y705) protein expression, with the majority of these (919/1279; 71.9%) being positively correlated. Of these, *CX3CR1*, which codes for the CX3C chemokine receptor 1, exhibited the strongest positive correlation with pSTAT3(Y705) (rho = 0.36), though *CX3CR1* is not included in the COSMIC Cancer Gene Census. Interestingly, CX3CR1 and STAT3 have been reported to positively cooperate in modulating the immune interaction between monocytes and smooth muscle cells, indicating this observed signal may be tumor cell-autonomous. [35,36] When we further filtered using the COSMIC Cancer Gene Census, we found that expression of 51 known cancer genes is associated with pSTAT3(Y705) expression, with *STAT3* mRNA demonstrating the strongest positive correlation (rho = 0.35) among them. We and others have previously demonstrated that STAT3 overexpression is an early event in HNSCC carcinogenesis, where STAT3 is upregulated and constitutively activated in cancer patients relative to healthy controls. [37] Furthermore, STAT3 expression is associated with disease stage, nodal status, tumor size, relapse-free survival, and overall survival in early-stage oral SCC. [38] While it was unsurprising to detect a correlation between *STAT3* mRNA and STAT3 pathway activation, we observed an unexpected correlation between pSTAT3(Y705) and *caspase-8* expression (rho = 0.28), as well as *caspase-10* expression (rho = 0.34). We sought to validate the *caspase-8*/pSTAT3(Y705) relationship in HNSCC cells and observed that exogenous overexpression of caspase-8 led to marked upregulation of pSTAT3(Y705) expression. We observed no upregulation of total STAT3 following overexpression of caspase-8, indicating that STAT3 mRNA overexpression and caspase-8 overexpression may be independent mechanisms of pSTAT3(Y705) upregulation. While it has been demonstrated that STAT3 inhibition leads to caspase-8-mediated apoptosis in several model systems [39–41], non-canonical caspase-8 signaling may alternatively lead to activation of NF-κB signaling [42], a pathway that exhibits extensive cross-talk with STAT3. [43] These findings suggest that this type of analysis may uncover unanticipated biology that contributes to oncogenic signaling.

We next analyzed whole exome sequencing data from the TCGA to identify any individual genes for which non-synonymous mutation was statistically associated with STAT3 activation in HNSCC tumors. When applying Student’s t-test, we found many mutant genes with an apparent statistical association with pSTAT3(Y705) expression. Subsequent analysis indicated these were due to in inappropriate application of the t test (2/206, < 1% in several cases), and a failure to screen low-frequency mutations. Application of the more appropriate Wilcoxon test failed to identify any significant association between somatic mutation and pSTAT3(Y705) expression (defined as q < 0.1). These findings indicate first that caution should be taken when interrogating large -omics data sets for identification of putative biomarkers, and secondly that larger cohorts will be required to identify downstream signaling consequences of infrequent alterations.

Promoter methylation or copy number alteration represent additional prominent mechanisms underlying oncogenic signaling in HNSCC. [44,45] We found that neither promoter hypermethylation nor copy number alteration for any individual gene was correlated with pSTAT3(Y705) expression, suggesting little direct contribution of gene silencing to STAT3 pathway overactivation. Alternatively, this may indicate that our methodology is too stringent to detect effects of these particularly complex biologic events. For example, we recently reported that promoter hypermethylation of the *PTPRT* gene is strongly associated with down-regulation of *PTPRT* mRNA, which in turn is associated with pSTAT3(Y705) overexpression. [13] In the present study, the absence of a direct statistically significant correlation between *PTPRT* methylation and pSTAT3(Y705) expression may be reflective of the noise and complexity of the many additional biologic steps between these two events rather than a true lack of association. Nevertheless, our analyses implicate many alterations, especially somatic mutation or alteration of mRNA expression, as potential mechanisms of STAT3 pathway activation in HNSCC. Further investigation may ultimately lead to deeper understanding of HNSCC biology as well as the identification of putative biomarkers of response to STAT3 inhibitors.

## Supporting Information

S1 Fig Spearman correlations for the 12 genes for which mRNA is most highly correlated with pSTAT3(Y705) expression. q < 0.005 for all depicted.
(TIF)

S2 Fig Apparent significant T tests suggesting correlation between somatic mutations and pSTAT3(Y705) expression are artifacts of low mutation frequency for any single gene. (TIF)

S3 Fig Hypermethylation of individual genes does not significantly correlate with pSTAT3(Y705) expression. A tail strength analysis indicates a lack of significant correlation between hypermethylation of any individual gene analyzed and pSTAT3(Y705) expression.
(TIF)

S1 Table mRNA expression correlates with STAT3 phosphorylation for 1279 genes. (XLSX)

S2 Table mRNA expression of known cancer genes significantly correlates with pSTAT3(Y705) protein expression. Genes are listed in order of descending rho value. Upregulation or downregulation for each indicated gene denotes the percentage of tumors with mRNA expression greater or less than two standard deviations from the mean (Z-score), respectively. Bars indicate relative up/downregulation frequency across genes.
(XLSX)
