# Supplementary figures and images for: Genomic and Transcriptomic Alterations Associated with STAT3 Activation in Head and Neck Cancer
Source: PLoS One. 2016 Nov 17;11(11):e0166185. doi: 10.1371/journal.pone.0166185 (PMC5113908; doi:10.1371/journal.pone.0166185)

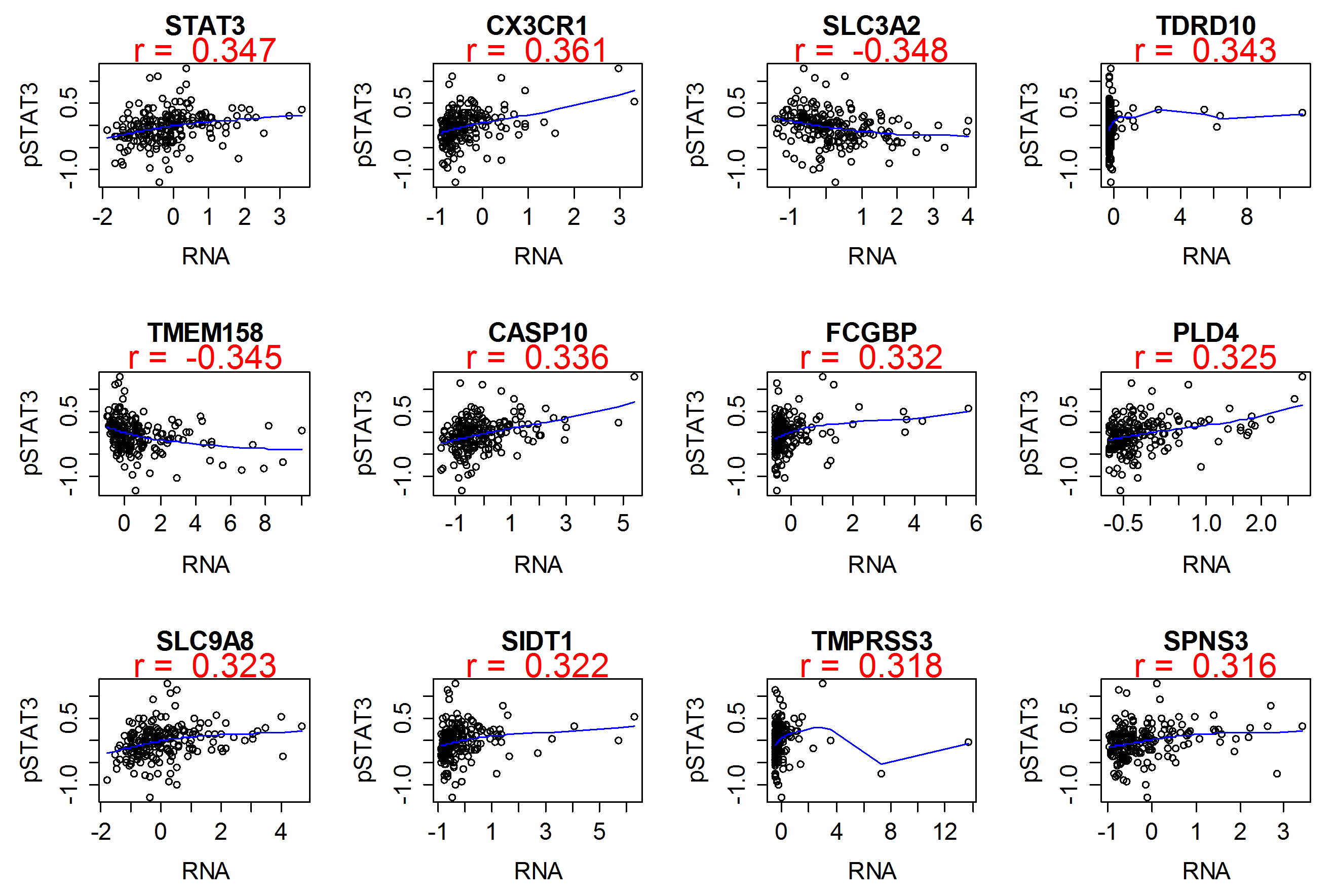

Supplement: S1 Fig — q < 0.005 for all depicted. (TIF) [file pone.0166185.s001.TIF]

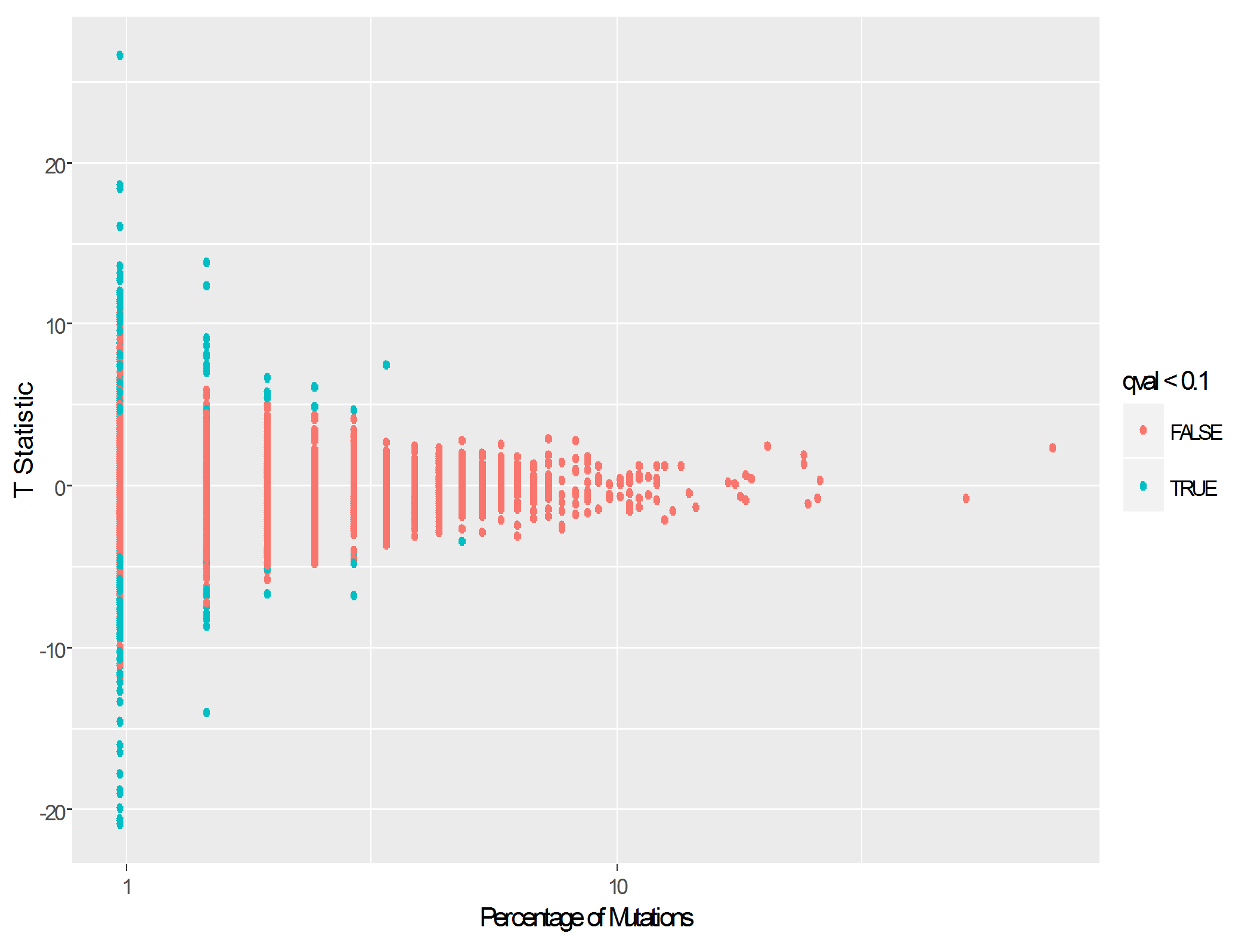

Supplement: S2 Fig — (TIF) [file pone.0166185.s002.TIF]

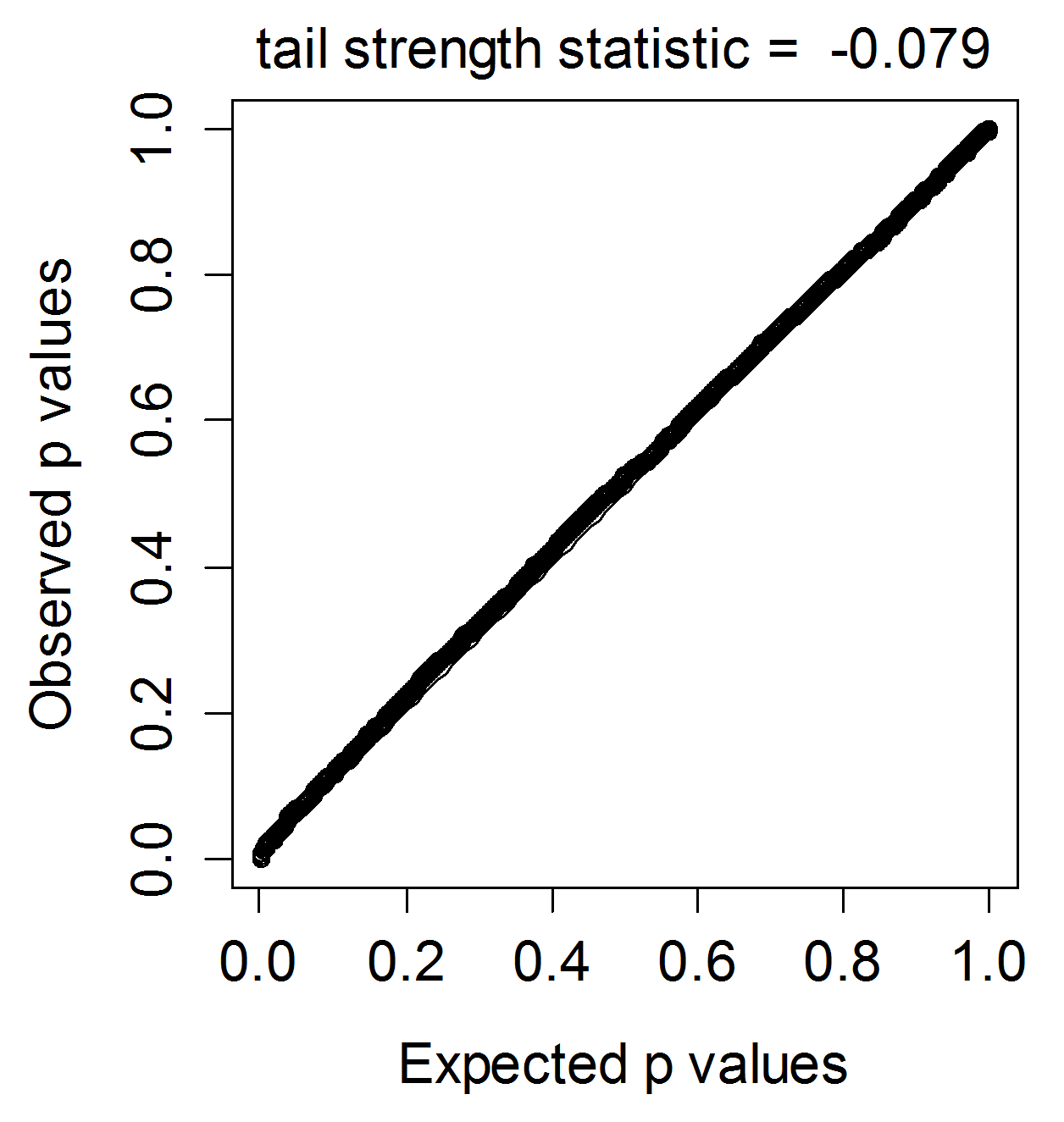

Supplement: S3 Fig — A tail strength analysis indicates a lack of significant correlation between hypermethylation of any individual gene analyzed and pSTAT3(Y705) expression. (TIF) [file pone.0166185.s003.TIF]
